# Artificial Intelligence in the Assessment of Males with Chronic Pelvic Pain Syndrome: An Up-to-Date UPOINTS-Based Narrative Mapping Review

**DOI:** 10.3390/diagnostics16152479

**Published:** 2026-08-06

**Authors:** Ali Talyshinskii, Fatima Kudakova, Olga Staroseltseva, Nariman Gadzhiev, Bhaskar Kumar Somani

**Affiliations:** 1 Department of Urology and Andrology, Genome Clinic, Astana 010000, Kazakhstan; urovator@gmail.com; 2 Department of Functional Urology, Saint-Petersburg State University Hospital, Saint Petersburg 191023, Russia; kudakova@yandex.ru (F.K.); o.staroseltseva@gmail.com (O.S.); nariman.gadjiev@gmail.com (N.G.); 3 Department of Urology, University Hospital Southampton NHS Trust, Southampton SO16 6YD, UK

**Keywords:** CPPS, pelvic pain, UPOINTS, AI

## Abstract

**Background/Objectives:** Male chronic pelvic pain syndrome (CPPS) is a heterogeneous condition involving overlapping urinary, psychosocial, organ-specific, infectious, neurological, myofascial, and sexual phenotypes. This complexity limits the effectiveness of routine symptom assessment and empirical treatment strategies. Artificial intelligence (AI) may support more reproducible interpretation, differential diagnosis, phenotyping, and personalized management. This up-to-date narrative mapping review aimed to identify AI-assisted approaches that are directly or indirectly relevant to the assessment of males with CPPS, classify them according to UPOINTS phenotypic domains and clinical functions, and critically discuss the extent to which current evidence is disease-specific or extrapolated from related conditions. **Methods:** A literature search was performed in PubMed/MEDLINE, the Cochrane Library, and Google Scholar from database inception to May 2026 using terms related to male CPPS, UPOINTS domains, diagnosis, treatment, prognosis, digital solutions, artificial intelligence, machine learning, deep learning, natural language processing, computer vision, and decision support. Studies were included if they described AI-based or AI-adjacent computational approaches relevant to male CPPS or to related conditions important for UPOINTS-based phenotyping, differential diagnosis, or phenotype-specific assessment. **Results:** Available evidence remains fragmented and is largely extrapolated from related urological, chronic pain, pelvic floor, infectious, neurological, and sexual medicine conditions. AI applications were most developed in urinary and organ-specific domains, including uroflowmetry analysis, bladder volume assessment, cystoscopy, prostate imaging, urinary biomarkers, and differential diagnosis of lower urinary tract disorders. AI tools also showed potential for infection detection, psychosocial screening, chronic pain monitoring, neuroimaging-based phenotyping, pelvic floor dysfunction assessment, and evaluation of sexual dysfunction. However, male-CPPS-specific validation remains limited. **Conclusions:** AI has promising potential to improve differential diagnosis, multidomain phenotyping, and individualized management in males with CPPS. Current evidence is mainly translational and hypothesis-generating. Future studies should focus on prospective male-CPPS-specific cohorts, external validation, explainable multimodal models, and integration of AI tools into clinically meaningful, patient-centered workflows.

## 1. Introduction

Current guidelines are moving away from an organ-focused interpretation and consider male chronic pelvic pain syndrome (CPPS) as a condition with a multifactorial and phenotypically variable nature [1]. In real-life practice, males with CPPS often have a combination of several overlapping phenotypic components, making differential diagnosis, symptom stratification, and selection of personalized multimodal therapy particularly challenging [2]. This clinical complexity explains why the traditional strategy based primarily on routine symptom assessment and empirical pharmacotherapy is often insufficient [3]. Against this backdrop, artificial intelligence (AI) is of particular interest as a tool for analyzing complex chronic conditions characterized by multiple data sources and significant phenotypic variability [4]. AI-assisted solutions have been shown to improve the reproducibility of interpretation, enhance clinical pattern recognition, support differential diagnosis, and enhance risk stratification across various urological entities [5]. However, despite rapidly growing interest in AI in urology, its precise role in male CPPS remains unclear. This up-to-date narrative mapping review aimed to identify AI-assisted approaches that are directly or indirectly relevant to the assessment of males with CPPS, classify them according to UPOINTS phenotypic domains and clinical functions, and critically discuss the extent to which current evidence is disease-specific or extrapolated from related conditions.

## 2. Materials and Methods

This article was designed as an up-to-date narrative mapping review rather than a systematic review or meta-analysis. The purpose was to identify and organize current artificial intelligence (AI)-assisted approaches that may be directly or indirectly relevant to the assessment of males with chronic pelvic pain syndrome, using the UPOINTS phenotypic framework as the organizing structure.

A literature search was performed in PubMed/MEDLINE, the Cochrane Library, and Google Scholar from database inception to May 2026. Search terms included combinations of condition-related, domain-related, and AI-related keywords: “male chronic pelvic pain,” “chronic prostatitis/chronic pelvic pain syndrome,” “CPPS,”, “UPOINTS,” “pelvic pain,” “lower urinary tract symptoms,” “uroflowmetry,” “bladder pain,” “prostatitis,” “urinary tract infection,” “sexually transmitted infection,” “central sensitization,” “chronic pain,” “pelvic floor dysfunction,” “myofascial pelvic pain,” “erectile dysfunction,” “sexual dysfunction,” “artificial intelligence,” “machine learning,” “deep learning,” “natural language processing,” “computer vision,” “large language model,” “prediction model,” “classification,” “segmentation,” and “decision support.” Boolean operators were used to combine CPPS-related terms with AI-related terms and with terms corresponding to individual UPOINTS domains. Additional relevant articles were identified by manual screening of the reference lists of selected publications and recent reviews.

Studies were considered eligible if they described the development, validation, or clinical application of AI-based methods relevant to at least one UPOINTS domain in males with CPPS or in a clinically related condition important for phenotype-specific assessment or differential diagnosis. Eligible AI-based approaches included machine learning, deep learning, natural language processing, computer vision, large language models, automated classification, automated prediction, image segmentation, signal interpretation, and decision-support models. Studies were included when their clinical task was relevant to CPPS assessment, including urinary phenotype evaluation, psychosocial screening, exclusion of organ-specific urological pathology, infection-related differential diagnosis, neurological or central sensitization phenotyping, pelvic floor dysfunction assessment, or sexual-domain characterization.

Studies were excluded if they did not include an AI-based or computational analytic component, were not clinically relevant to any UPOINTS domain, focused only on general digital health without algorithmic interpretation, or had no plausible relevance to male CPPS assessment, phenotyping, differential diagnosis, or management. Non-English articles, conference abstracts without sufficient methodological detail, editorials without original data, and articles for which the full text was unavailable were not prioritized for inclusion.

Titles, abstracts, and full texts of potentially relevant publications were screened for clinical relevance to male CPPS and to the UPOINTS framework. Because direct male-CPPS-specific AI evidence was limited, selected studies were interpreted according to three levels of relevance: direct CPPS evidence, defined as studies developed or validated in male CPPS or CP/CPPS populations; CPPS-adjacent evidence, defined as studies in conditions directly relevant to UPOINTS phenotyping or differential diagnosis; and translational or exploratory evidence, defined as broader AI applications in chronic pain, pelvic floor dysfunction, psychosocial assessment, sexual medicine, infection-related diagnostics, or urological imaging with conceptual relevance to CPPS.

Because this article was designed as a narrative mapping review, no formal PRISMA-based systematic review workflow, protocol registration, quantitative study selection counts, or meta-analysis was performed. Instead, we used a structured evidence-mapping framework to transparently organize the literature according to clinical relevance to male CPPS and UPOINTS domains. This framework included identification of potentially relevant AI-related publications; screening for relevance to male CPPS assessment, UPOINTS phenotyping, or differential diagnosis; exclusion of studies without an AI-based analytic component or plausible CPPS relevance; classification of included studies as direct CPPS evidence, CPPS-adjacent evidence, or translational/exploratory evidence; and narrative synthesis within the corresponding UPOINTS domains.

The selected literature was narratively synthesized according to the UPOINTS domains: urinary, psychosocial, organ-specific, infection, neurologic/systemic, tenderness/myofascial, and sexual dysfunction. For each domain, we summarized the clinical focus, dominant AI-enabled tasks, typical input data, expected clinical value, interpretive cautions, and priorities for future validation. No quantitative synthesis was performed because of substantial heterogeneity in study populations, AI tasks, input data, reference standards, validation strategies, and reported outcomes.

A formal risk-of-bias assessment was not performed because of the narrative mapping design and the substantial heterogeneity of included studies. Reported performance metrics such as AUC, accuracy, sensitivity, specificity, or F1 score were therefore interpreted as indicators of technical model performance rather than as evidence of clinical readiness for male CPPS care.

## 3. Results

**Figure 1:**
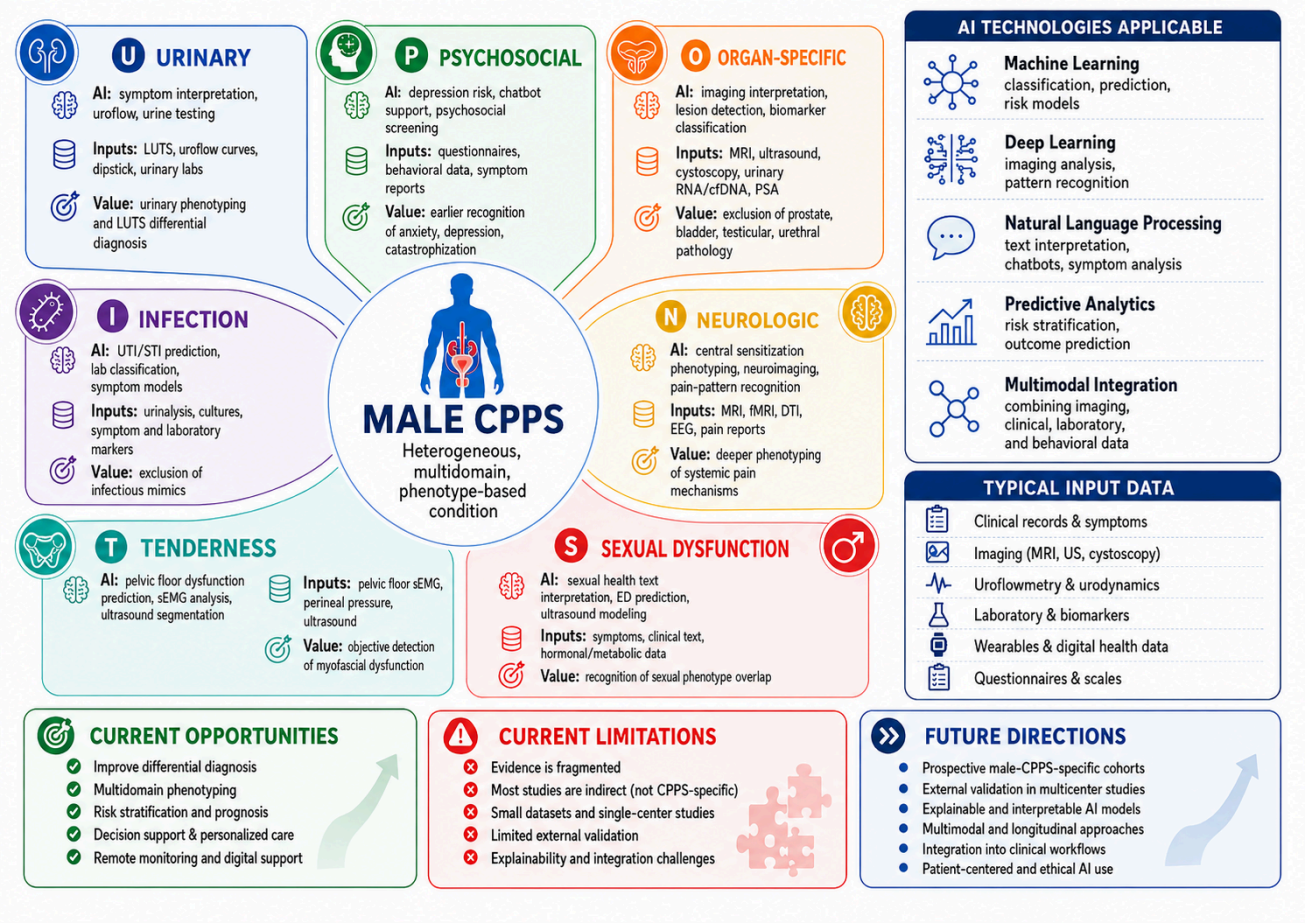
UPOINTS-based framework of artificial intelligence applications potentially relevant to assessment, phenotyping, and differential diagnosis in males with chronic pelvic pain syndrome. Note: Several AI applications depicted in this framework remain translational or hypothesis-generating and have not yet been clinically validated in male CPPS-specific cohorts.

The identified literature was heterogeneous and was not limited to studies performed exclusively in male CPPS cohorts. Accordingly, the evidence was synthesized according to its clinical relevance to UPOINTS domains rather than disease identity alone [6]. AI studies from adjacent conditions were included only when they addressed phenotype-specific assessment or differential diagnosis relevant to males with CPPS (Figure 1). Therefore, the following sections should be interpreted as a clinical evidence map rather than a summary of AI models validated exclusively in male CPPS.

### 3.1. Domain U

The U (urinary) domain reflects one of the most common associated symptom patterns, including a combination of pain with storage or voiding symptoms, occurring in 60–72% of patients [7]. Current guidelines indicate the need for a comprehensive differential diagnosis, including a thorough anamnesis and instrumental methods, including endoscopy, due to the multitude of organic and functional conditions with related symptoms. Thus, the use of generative neural networks for prompt patient counseling and the initial assessment of complaints and anamnesis shows promise. Nino et al. described GPT’s capability for responses on male lower urinary tract symptoms (LUTS) suggestive of benign prostate enlargement (BPE) when compared to two reference resources with an F1 score of 0.79, a median quality score of 4, and a good level of agreement between examiners [8]. Bae et al. evaluated the performance of ChatGPT-4o in estimating International Prostate Symptom Score (IPSS) and Overactive Bladder Symptom Score (OABSS) based on patients’ natural language descriptions and full outpatient records was evaluated, with AUCs of 0.81 and 0.91, respectively [9]. Along with complaint analysis, various attempts to integrate AI into urinalysis were described, also reflecting some conditions leading to LUTS and potentially suitable for use in diagnosing related males with CPPS. Kim et al. investigated the effectiveness and accuracy of an AI-based program in automatically interpreting urine test strips using mobile phone cameras; the AI algorithm demonstrated 98.7% accuracy in detecting urinary nitrite and 97.3% accuracy in detecting urinary glucose [10].

Within this domain, the most studied use of AI relates to the uroflowmetry curves analysis [11]. Şığva et al. developed an AI-assisted framework for the automated evaluation of uroflowmetry data in patients presenting with lower urinary tract symptoms with an accuracy of 92.5%, sensitivity of 90.0%, specificity of 94.0%, and an AUC-ROC of 0.96 [12]. Matsukawa et al. established an artificial intelligence diagnostic system for lower urinary tract function in men, revealing detrusor underactivity (DU) and bladder outlet obstruction (BOO) with specificity of 88.7% and 84.7%, respectively, outperforming urologists’ opinion [13]. Tsai et al. described at-home vibration-based uroflowmetry empowered by AI with high accuracy, with all three indicators (precision, recall, and F1 score) surpassing 0.97 [14]. Equally promising is the use of AI in the automatic determination of post-void urine volume, which can reduce analysis time and interobserver variability, especially with the advent of a portable ultrasound (US) option [15]. Jeong et al. prospectively investigated whether bladder volume measured using deep learning AI is more accurate than that measured using conventional methods when using a portable US bladder scanner and revealed greater accuracy for AI in their selected cohort on internal validation [16]. Finally, there are several models proposed for endoscopic analysis of the lower urinary tract that contribute to this phenotype [17]. For example, Eun et al. proposed an AI model to recognize and guide the operator to the exact stenosis area during endoscopic surgery in patients with urethral strictures characterized by an average final sensitivity value of 0.96 [18].

Overall, the evidence in the urinary domain should be interpreted mainly as CPPS-adjacent rather than direct male-CPPS-specific evidence. The reviewed AI models address LUTS interpretation, uroflowmetry analysis, bladder function classification, urinalysis interpretation, bladder volume estimation, and endoscopic recognition of urethral pathology, all of which are clinically relevant to urinary phenotype assessment and differential diagnosis in males with CPPS. However, these models were generally not developed or validated specifically in male CPPS cohorts; therefore, their current role is best understood as supportive for urinary-domain clarification rather than as disease-specific CPPS diagnostic tools.

### 3.2. Domain P

The psychosocial domain in the UPOINTS system is especially problematic and characterized by a direct impact on the effectiveness of the entire therapy and patients’ perception of their status, increasing pain reaction and worsening quality of life. It should be noted that clearly distinguishing this domain from similar chronic pain diseases is difficult, which explains the sporadic nature of such studies and allows for the description of other studies on the use of AI among patients with chronic pain. Shen et al. developed a predictive model to ascertain the likelihood of depression among males with CPPS; it demonstrated an AUC of 0.864 and 0.911 in the training and validation cohort, respectively [19]. Piette et al. described a randomized trial in patients with chronic back pain, where all patients received 10 weeks of treatment, but in the AI group, a reinforcement learning-based system analyzed patient feedback via short automated calls and decided which format of contact was needed that week: a full 45 min conversation with a therapist, a short 15 min session, or a personalized voice message. According to the results, such a system was no less effective than cognitive behavioral therapy (CBT) with a doctor, reducing time costs by 48% [20]. Hauser-Ulrich et al. described mobile health intervention utilizing a fully automated text-based health care chatbot (TBHC) and revealed mainly positive feedback and valuable suggestions from patients [21]. Along with the work described by Li et al. performed meta-analysis of AI-based conversational agents for mental health and stated that such technology significantly reduces symptoms of depression and distress [22]. Similarly, in another meta-analysis Zhong et al. revealed that AI-chatbots demonstrate significant reductions in depression and anxiety, whereas therapeutic effects begin at four weeks and intensify after eight weeks of their use [23]. Finally, Jamison et al. described a mHealth app classifying patients pain data to determine pain catastrophizing, which is not related to AI but is easily adaptable to this technology [24].

The psychosocial domain contains limited direct CPPS-related AI evidence, particularly the model predicting depression in patients with CP/CPPS, while most other data are extrapolated from broader chronic pain or mental health populations. These studies are relevant because depression, anxiety, distress, and catastrophizing can influence pain perception, treatment response, and quality of life in males with CPPS. Nevertheless, current AI applications in this domain should be considered supportive screening or self-management tools rather than validated CPPS-specific psychosocial decision-support systems.

### 3.3. Domain O

The O (organ-specific) domain reflects the probable involvement of a urogenital organ in the structure of the general CPPS. According to the international nomenclature, within the framework of male CPPS, primary prostate pain syndrome is distinguished; Primary bladder pain syndrome; primary urethral pain syndrome; primary penile pain syndrome; primary scrotal pain syndrome with subtypes of primary testicular pain syndrome and primary epididymal pain syndrome; post-vasectomy scrotal pain syndrome; and primary perineal pain syndrome [1]. Therefore, when analyzing AI tools for the diagnosis and treatment of males with CPPS within the “O” domain, it is necessary to describe approaches to exclude organic pathologies. Along with examples for the “U” domain, Chang et al. evaluated the feasibility of real-time AI integration during clinic cystoscopy and transurethral resection of bladder tumor (TURBT) with per-frame tumor specificity of 98.8% and a median error rate of 3.6% [25]. Meng et al. assessed the diagnostic performance of cfDNA hotspot-driven machine learning models for noninvasive bladder cancer (BLCA) detection with specificity of 71% and 92% for early (low-grade Ta and T1) and advanced (high-grade T1 and muscle-invasive) disease, respectively [26]. Kaya et al. assessed the capability of deep learning models for distinguishing prostatitis and prostate cancer lesions on MRI and found performance metrics of >90% regardless of imaging sequences [27]. Goh et al. developed a diagnostic framework integrating ML with non-invasive urinary RNA biomarker profiles obtained from DRE-free whole urine with AUC of 0.99 for the Gradient Boosting model in detecting PCa [28]. Fang et al. developed an US-driven clinical deep learning radiomics (CDLR) model for stratifying the risk of testicular masses showing AUC of 0.835 in external validation when distinguishing testicular tumors from non-tumorous lesions [29]. Feng et al. validated AI-enhanced three-dimensional sonourethrography that provided detailed views of the corpus spongiosum fibrosis from multiple perspectives [30].

The evidence in the organ-specific domain is predominantly CPPS-adjacent and is most relevant for differential diagnosis rather than direct CPPS phenotyping. AI models for cystoscopy, prostate MRI, urinary biomarkers, testicular ultrasound, and urethral imaging may help exclude competing urological pathology that can mimic or contribute to organ-specific pelvic pain symptoms. However, these tools do not model CPPS itself, and their value in CPPS care depends on future validation as structured exclusion or triage tools within male pelvic pain diagnostic pathways.

### 3.4. Domain I

Although CPPS by definition refers to conditions in which an infectious origin is not considered a proven underlying mechanism, domain I (Infection) may be positive in some of these patients. Accordingly, a positive infection domain among males with CPPS does not necessitate a revision of the diagnosis to chronic bacterial prostatitis, but rather indicates a possible concomitant or partial infectious–inflammatory contribution to the clinical picture. Given this, the corresponding AI models are particularly relevant both for verifying this phenotype and for initially excluding infectious genesis of complaints [31]. Soe et al. developed an online symptom checker (iSpySTI.org) based on ML for the sexually transmitted infection (STI) diagnosis, which performed well for most male conditions, with the AUC ranging from 0.81 to 0.95 [32]. Similarly, Xu et al. developed a web-based HIV and STI risk prediction tool using machine learning algorithms (MySTIRisk), performed at an acceptable or excellent level on testing datasets (AUC for HIV = 0.78; AUC for syphilis = 0.84; AUC for gonorrhea = 0.78; AUC for chlamydia = 0.70) [33]. Flores et al. used ML models to improve UTI diagnostics with AUC between 0.81 and 0.88 [34]. Ozkan et al. developed AI models to support the diagnosis of UTI with complex symptoms, among which artificial neural network (ANN) had the highest accuracy result of 98.3% and were mainly based on pollakiuria, suprapubic pain symptoms and hematuria [35]. Favresse et al. evaluated six ML algorithms for their ability to predict UTIs based on 5 definitions including pyuria and cultural outcomes. According to results, CatBoost Classifier achieved an AUC of 92.0–94.7% depending on the UTI definition [36]. Rodríguez Del Águila et al. used ML to optimize UTI detection using non-microbiological laboratory parameters, thereby reducing unnecessary cultures and expediting diagnosis with accuracy for Random Forest of 82.2%, whereas among the most relevant predictive variables were the presence of leukocytes and nitrites in the urine dipstick test, along with elevated white cells count, monocyte count, lymphocyte percentage in blood and creatinine levels [37].

The infection domain is best interpreted as CPPS-adjacent evidence focused on exclusion of infectious mimics or identification of infection-related contributors. AI models for STI and UTI prediction may be clinically useful in males presenting with pelvic pain and overlapping urinary or sexual symptoms, particularly when infection is part of the differential diagnosis. However, these models do not establish CPPS-specific infectious phenotypes and should be integrated with clinical assessment, urinalysis, culture results, and guideline-based diagnostic reasoning.

### 3.5. Domain N

The N (neurological) domain should be considered a manifestation of central sensitization and a systemic–neurobiological variant of pain syndrome, characterized by pain outside the lower abdomen, genitals, and pelvis, as well as chronic overlapping pain conditions (IBS, fibromyalgia, chronic fatigue syndrome), which justifies its separate identification and distinction from other domains. According to the MAPP Research Network Study, among 424 enrolled patients, 25% reported pelvic pain only, and 75% reported pain beyond the pelvis, of which 38% reported widespread pain. Participants with a greater number of pain locations had greater non-pelvic pain severity (*p* < 0.0001), sleep disturbance (*p* = 0.035), depression (*p* = 0.005), anxiety (*p* = 0.011), psychological stress (*p* = 0.005), negative affect scores (*p* = 0.0004), and worse quality of life (*p* ≤ 0.021) [38]. Given the diversity of possible manifestations, the use of AI is a relevant direction for diagnosis and treatment in this case, allowing for personalized phenotyping of CPPS. Bagarinao et al. using MRI data from the Trans-MAPP Research Network and linear support vector machine (SVM) algorithm to differentiate cerebral gray matter images between patients with CPPS and controls and found that activity change in primary somatosensory cortex, pre-supplementary motor area, hippocampus, and amygdala enabled the classification with 73% overall accuracy to predict the presence of CPPS [39]. According to Lan et al., patients presented decreased resting-state functional connectivity (rs-FC) between the basal ganglia (BG) subregions and right middle cingulate cortex on MRI, and this was correlated with pain and urinary symptoms, whereas an AUC of 0.943 was achieved for the classification of CPPS patients and healthy males using SVM [40]. Zeng et al. proposed a ML-based approach utilizing resting-state functional MRI and diffusion tensor imaging (DTI) to enhance the clinical diagnostic efficiency of fibromyalgia (FM) with an AUC of 0.98 [41]. Zheng et al. used unsupervised machine learning to refine the Central Sensitization Inventory (CSI) threshold value among patients with chronic back pain, achieving sensitivity and specificity of 0.76 and 0.76 for 35 points cut-off [42]. Chen et al. used Bayesian Additive Regression Trees, an ML algorithm, to develop predictive models for pain severity, pain interference, and quality of life (QOL) among young adults with IBS with AUC from 0.753 to 0.981 [43]. Levitt et al. used ML to diagnose chronic lumbar radiculopathy and identify corresponding patterns using electroencephalography (EEG) and achieved an AUC of 0.826 [44]. On the other hand, Slepian et al. developed Solace, an expert-trained generative AI conversational agent for patients with chronic pain, who rated its usability as excellent and demonstrated improvements in anxiety, pain interference, kinesiophobia, and pain resilience [45]. Amidei et al. used GPT-4 to analyze the text of 43 patients with fibromyalgia to assess pain severity and disability, demonstrating a weighted percentage agreement of >0.95 compared with experts [46]. In this context, the scoping review by Souza et al. is informative, describing chatbots for collecting health information, providing emotional support, self-management exercises, psychological treatment, and training specialists [47].

The neurological domain includes some of the most conceptually disease-relevant AI evidence, including neuroimaging-based studies in CPPS populations, but it also contains broader translational evidence from fibromyalgia, chronic low back pain, irritable bowel syndrome, and other chronic pain conditions. These studies are relevant because they address central sensitization, widespread pain, pain interference, and neurobiological heterogeneity, which are important in systemic or neurologic CPPS phenotypes. At present, however, these approaches remain exploratory and require prospective validation linking neuroimaging, symptom profiles, UPOINTS domains, and clinically meaningful outcomes in male CPPS.

### 3.6. Domain T

The T (tenderness) domain corresponds to the presence of a local myofascial component of pain, manifested by pelvic floor myalgia or trigger points of the pelvic floor and adjacent muscles, detected during physical examination. This characteristic fundamentally distinguishes this pattern from the U and N domains, which are characterized by LUTS or extrapelvic pain, respectively. According to cohort studies, the frequency of the T domain varies from 40 to 56.4%, and the frequency of the painful trigger localization also varies [48,49]. Thus, in the NIH Chronic Prostatitis Cohort Study, among 384 males with CPPS, 51% had at least one tender area on examination versus 7% in asymptomatic controls; the most common were prostate tenderness (41%), followed by the external pelvic floor (13%), internal pelvic floor (14%), and suprapubic area (9%) [50]. In terms of differential diagnosis, it is necessary to consider the possibility of entrapment syndromes, which can be both a consequence and a cause of muscle hypertonicity. This pathophysiologically aligns such cases with the N domain and can create overlap between the T and N domains. Given the frequency, the possibility of instrumental assessment, and the overlap with other domains, the use of AI appears particularly attractive, allowing for the specific detection of pelvic floor dysfunction (PFD) and reducing both the rate of underdiagnosis and the discrepancy in mapping between specialists. It should be noted that most studies describe a mixed or female population, which allows only a hypothetical assessment of the usefulness of such solutions among males. Li et al. described an ML-based prediction model for myofascial pelvic pain syndrome (MPPS) with an AUC of 0.956 for Random Forest, supporting early diagnosis and enabling personalized clinical decision-making [51]. Chen et al. developed an AI–Diagnostician–PFD diagnostic model to derive AI reference ranges for pelvic floor surface electromyography (sEMG) parameters which achieved 11% higher AUC compared to Glazer standard [52]. Hwang et al. described ML-empowered noninvasive PFM strength quantification by leveraging extracorporeal surface perineal pressure (ESPP) [53]. El-Sayegh et al. proposed a PFM contraction quality assessment system combining contraction detector and maximum contraction classifier based on AI and predicting the strength rating within a ±1 scale point with 97% accuracy [54]. García-Mejido et al. applied a convolutional neural network (CNN) for identifying different organs of the pelvic floor in the midsagittal plane via dynamic ultrasound and demonstrating the highest segmentation accuracy (83%) for levator ani muscle [55]. Bowness et al. published a scoping review of the evidence on the application of artificial intelligence for optimization and interpretation of the sonographic image, and visualization of needle advancement and injection of local anesthetic, in particular cNerve (GE Healthcare) and Smart Nerve (Mindray) systems incorporated into the manufacturers’ ultrasound machines and segmenting peripheral nerves to produce a (yellow) color overlay, which is expected to aid in the differential diagnosis of males with CPPS in the future [56].

The tenderness domain is highly clinically relevant to male CPPS because pelvic floor myalgia and trigger points are frequent components of the syndrome, but the current AI evidence remains mostly extrapolated from mixed or female pelvic floor dysfunction cohorts. AI-based analysis of pelvic floor sEMG, perineal pressure signals, contraction quality, ultrasound imaging, and nerve visualization may improve objective assessment of myofascial dysfunction and reduce interobserver variability. However, male-CPPS-specific validation is still needed before these tools can be used for phenotype-guided diagnosis or treatment planning in males with pelvic pain.

### 3.7. Domain S

In recent years, the addition of a sexual (S) domain to the classic UPOINTS scheme has become relevant and covers specific symptoms, including erectile dysfunction (ED), ejaculatory dysfunction, orgasmic dysfunction, and decreased libido. According to a dedicated meta-analysis, the overall incidence of sexual dysfunction among males with CPPS is 62%, while the prevalence of erectile dysfunction and premature ejaculation was 29% and 40%, respectively [57]. In addition, the presence of sexual dysfunction is associated with a more unfavorable psychoemotional state and symptom severity [58]. The literature contains ample references to the use of AI in various aspects of sexual dysfunction, potentially applicable to males with CPPS in conjunction with other models for comprehensive analysis and treatment planning. Harrison et al. used a natural language processing (NLP) approach to identify sexual health information in clinical text, achieving 0.89–1 Cohen’s kappa values when compared to expert conclusions [59]. Ran et al. described the capabilities of GPT v4.0 in answering 30 different questions regarding sexual complaints and found a reasonable response in 86.7% of cases [60]. Chen et al. developed an ML-based prediction model for ED based on age, obesity, cardiovascular risk factors, prostate-related diseases, and socioeconomic status and achieved an AUC of 0.887 for XGBoost [61]. Yu et al. described prediction model based on Light Gradient Boosting Machine (LGBM) for testosterone deficiency in adult males with body mass index (BMI), high-density lipoprotein and muscle quality index (MQI) being the most influential factors and achieved AUC of 0.746 [62].

The sexual dysfunction domain is clinically relevant to male CPPS, but current AI evidence is largely extrapolated from the adjacent andrology and sexual medicine literature. AI tools for sexual health text interpretation, erectile dysfunction prediction, testosterone deficiency risk modeling, and patient-facing question answering may help identify sexual symptoms that overlap with pain, psychosocial burden, and urinary complaints. However, these approaches should not yet be considered CPPS-specific sexual phenotype models, and future studies should integrate sexual outcomes with pain severity, psychosocial variables, and UPOINTS-based clinical phenotyping in male CPPS cohorts.

## 4. Discussion

Male CPPS is a clinically heterogeneous syndrome characterized by variable combinations of pain, urinary, sexual, myofascial, psychosocial, organ-specific, infectious, and neurological manifestations, which explains the need for a multidisciplinary and phenotype-based approach [63]. This complexity makes AI conceptually relevant for this field, as AI may help integrate heterogeneous data sources, improve reproducibility of interpretation, and support stratification of patients according to clinically meaningful patterns [64] (Table 1). However, the findings of this up-to-date UPOINTS-based narrative mapping review show that the current evidence base remains fragmented and is still developing.

The available literature is concentrated mainly in areas where clinical information can be transformed into structured or quantitative inputs, particularly urinary, organ-specific, imaging-based, laboratory-based, and signal-based domains. In these settings, AI has been explored not only for differential diagnosis, but also for symptom interpretation, automated pattern recognition, risk prediction, and phenotype clarification. At the same time, evidence directly generated in male CPPS cohorts remains limited. Some disease-specific data exist, including AI-based approaches to psychosocial risk prediction and neuroimaging-based classification in CPPS populations, but much of the broader evidence comes from adjacent fields relevant to UPOINTS-based assessment rather than from CPPS-specific validation studies.

This indirectness is one of the central findings of the review. For example, infection-domain evidence is largely derived from AI studies focused on STIs and UTIs, while tenderness-domain evidence frequently comes from mixed or female pelvic floor dysfunction cohorts. Similarly, organ-specific and sexual-domain studies are clinically relevant for differential diagnosis and phenotype clarification, but they do not directly establish AI-assisted CPPS management strategies (Table 2). Therefore, the current literature should be interpreted primarily as translational and hypothesis-generating. It demonstrates where AI may support structured assessment of males with CPPS, but it does not yet justify disease-specific AI-driven diagnosis or treatment selection.

Taken together, these findings suggest that the most realistic role of AI in male CPPS at present is not autonomous clinical decision-making, but support for reproducible UPOINTS-based assessment, differential diagnosis, and multidomain phenotyping. The field now needs to move from isolated predictive models and extrapolated evidence toward explainable, externally validated, clinician-facing tools developed in prospective male-CPPS-specific cohorts. These considerations also require a more cautious interpretation of reported model performance, a clearer distinction between algorithmic benefit and the value of structured input data, and attention to practical, ethical, regulatory, and workflow barriers before AI can be integrated into routine CPPS care.

### 4.1. Most Promising Directions and Implementation Barriers

Based on the current evidence map, the most promising near-term AI directions for male CPPS are likely to be those that support structured assessment rather than autonomous diagnosis. These include automated interpretation of urinary symptoms and uroflowmetry patterns, AI-supported recognition of psychosocial vulnerability, decision-support tools for exclusion of organ-specific urological pathology, infection-oriented triage models, neurobiological or central sensitization phenotyping, objective pelvic floor function assessment, and integration of sexual dysfunction screening into multidomain CPPS evaluation. Among these areas, the most clinically actionable applications are those that can be embedded into existing UPOINTS-based workflows and help clinicians standardize phenotype recognition, identify patients requiring additional domain-specific evaluation, and support multidisciplinary care.

However, several barriers must be addressed before AI can be implemented in routine male CPPS care. From a workflow perspective, AI tools must be integrated into clinical pathways without increasing documentation burden or fragmenting the clinician–patient interaction. From a regulatory perspective, AI systems intended for diagnosis, risk stratification, or treatment support require transparent validation, performance monitoring, and clear definition of clinical responsibility. Ethical issues are also important, particularly because CPPS is a chronic pain condition associated with psychological distress, sexual symptoms, and sensitive patient-reported data. Therefore, privacy protection, explainability, bias assessment, informed use of AI-generated recommendations, and avoidance of over-reliance on nonvalidated algorithms should be considered essential requirements for future AI-assisted CPPS tools.

### 4.2. Critical Appraisal and Generalizability of the Current AI Evidence

The reported diagnostic or predictive performance of individual AI models should be interpreted cautiously. In several studies, high AUC, accuracy, sensitivity, specificity, or F1 score values reflect technical performance within a specific dataset rather than validated clinical utility in males with CPPS. Many models were developed in retrospective or single-center cohorts, used selected populations, relied on domain-specific reference standards, or lacked independent external validation. These factors may increase the risk of overfitting, spectrum bias, dataset shift, and reduced generalizability when models are applied to a heterogeneous CPPS population.

Another important limitation is indirectness. A considerable proportion of the reviewed evidence comes from LUTS, urological oncology, infectious disease, pelvic floor dysfunction, chronic pain, and sexual medicine rather than from male CPPS cohorts. Although these studies are clinically relevant to UPOINTS-based assessment and differential diagnosis, they should not be interpreted as direct evidence that AI improves diagnosis, phenotyping, or management of CPPS itself. In addition, few studies provide implementation-level outcomes, such as effects on clinical workflow, clinician decision-making, patient-centered outcomes, treatment selection, symptom improvement, or long-term follow-up. Therefore, the current AI evidence should be considered technically promising but clinically immature for male CPPS. Future studies should prioritize prospective CPPS-specific cohorts, transparent model reporting, external validation, calibration assessment, explainability, comparison with clinician performance, and evaluation of patient-centered outcomes before AI tools can be recommended for routine phenotype-guided CPPS care.

An additional consideration is that the apparent diagnostic or phenotyping benefit of AI may not be attributable solely to the algorithm itself. Some improvement may result from the use of structured, consistently defined, and systematically collected input data. This issue is particularly relevant in male CPPS, where routine clinical assessment is often affected by heterogeneous symptom descriptions, variable terminology, inconsistent physical examination findings, and differences in how urinary, psychosocial, organ-specific, infectious, neurological, myofascial, and sexual domains are documented. In this context, AI-based tools may improve performance partly because they impose a standardized data structure on a clinically heterogeneous syndrome.

### 4.3. Clinical Relevance and Potential Place in CPPS Care

From a clinical perspective, the most realistic near-term value of AI in male CPPS is not autonomous diagnosis or treatment selection, but support for more structured, reproducible, and phenotype-aware assessment. Within a UPOINTS-based workflow, AI tools may assist clinicians in several specific steps: interpretation of urinary symptoms and uroflowmetry patterns in the urinary domain; identification of psychosocial burden and pain-related vulnerability in the psychosocial domain; exclusion or triage of organ-specific urological pathology in the organ-specific domain; recognition of infectious mimics or contributors in the infection domain; characterization of widespread pain, central sensitization, or neurological overlap in the neurologic/systemic domain; objective assessment of pelvic floor dysfunction in the tenderness domain; and recognition of sexual dysfunction as a clinically relevant overlapping phenotype.

Therefore, the clinical contribution of the current evidence should be viewed as pathway-supportive rather than disease-defining. AI may help standardize the assessment of complex multidomain presentations, reduce interobserver variability, identify patients requiring domain-specific evaluation, and support multidisciplinary decision-making. However, the current literature does not yet support AI as a stand-alone diagnostic or therapeutic tool for male CPPS. Its future clinical value will depend on prospective validation in male CPPS cohorts, integration with guideline-based UPOINTS assessment, and demonstration that AI-assisted workflows improve clinically meaningful outcomes such as symptom burden, quality of life, treatment selection, patient adherence, and follow-up efficiency.

### 4.4. Original Contribution of This Review

The main original contribution of this review is the organization of fragmented AI evidence through a clinically familiar UPOINTS framework for male CPPS. Rather than presenting AI applications as isolated technological examples, this review links each AI task to a specific clinical function within CPPS assessment: urinary phenotype clarification, psychosocial burden recognition, exclusion of organ-specific pathology, infection-related differential diagnosis, neurological or systemic pain phenotyping, objective assessment of pelvic floor tenderness, and characterization of sexual dysfunction. This domain-based structure may help clinicians understand where current AI evidence is directly applicable, where it is only CPPS-adjacent, and where it remains purely translational.

This synthesis also identifies an important gap in the current literature: AI research relevant to male CPPS is developing faster in adjacent domains than in CPPS-specific cohorts. Therefore, the review provides a practical framework for future studies by indicating which AI tools should be validated prospectively in male CPPS populations and how they could be integrated into phenotype-guided clinical workflows. In this sense, the article is not intended merely as a catalogue of AI applications, but as a clinically structured roadmap for translating AI from adjacent urological, chronic pain, pelvic floor, infectious, neurological, psychosocial, and sexual medicine fields into CPPS-specific research and care.

## 5. Conclusions

Male CPPS is a heterogeneous multidomain condition with overlapping urinary, psychosocial, organ-specific, infectious, neurological, myofascial, and sexual phenotypes, making it a promising field for AI-assisted assessment and phenotype clarification. Current evidence suggests that AI may have its most realistic near-term role as a decision-support component of UPOINTS-based assessment, helping clinicians structure differential diagnosis, phenotype clarification, and domain-specific evaluation. However, most available data are derived from related conditions rather than male-CPPS-specific cohorts, and AI should not yet be considered a stand-alone diagnostic or treatment selection tool for male CPPS. Future research should focus on prospective male-CPPS-specific validation of explainable and multimodal AI tools, integration of patient-reported outcomes and longitudinal monitoring, and implementation within ethical, privacy-preserving, and clinically meaningful workflows.

## Figures and Tables

**Table 1:** UPOINTS-based conceptual map of artificial intelligence applications potentially relevant to assessment, phenotyping, and differential diagnosis in males with chronic pelvic pain syndrome.

UPOINTS Domain	Clinical Focus in Male CPPS	Dominant AI-Enabled Tasks	Typical Input Data	Expected Clinical Value	Evidence Status
Urinary	LUTS-associated phenotype, voiding/storage symptoms, exclusion of functional urinary disorders	Symptom interpretation, automated uroflowmetry analysis, bladder function classification, urine test interpretation, bladder volume estimation	Natural-language symptom descriptions, uroflowmetry curves, urine dipstick data, portable ultrasound signals, endoscopic visual data	Faster and more standardized assessment of urinary phenotype; support for differential diagnosis of LUTS-related conditions	Mostly CPPS-adjacent
Psychosocial	Depression, anxiety, catastrophizing, psychosocial burden	Risk prediction, mental health screening, conversational support, classification of psychosocial vulnerability	Questionnaire-derived variables, text-based interactions, symptom reports, behavioral feedback data	Earlier recognition of psychosocial burden and more structured phenotype-aware support	broader chronic pain and mental health AI evidence remains mostly translational
Organ-specific	Exclusion of competing urological pathology contributing to organ-related pain phenotype	Imaging interpretation, lesion classification, organ- level diagnostic support	MRI, ultrasound, cystoscopy images, urinary biomarkers, cfDNA/RNA profiles	Improved exclusion of prostate, bladder, testicular, or urethral pathology mimicking CPPS	mainly validated in organ-specific urological conditions rather than CPPS
Infection	Identification of infection-related phenotype or exclusion of infectious mimics	STI/UTI prediction, symptom checker models, laboratory-based diagnostic classification	Symptom questionnaires, urinalysis, culture-related parameters, blood and urine laboratory markers	Support for distinguishing inflammatory/infectious contributors from noninfectious CPPS	CPPS-adjacent evidence for infectious mimics
Neurologic	Central sensitization, widespread pain, neurobiological phenotype	Neuroimaging-based classification, pain phenotyping, symptom severity prediction, AI-assisted pain pattern recognition	Structural MRI, resting- state fMRI, DTI, EEG, pain reports, symptom variability data	Deeper phenotyping of central pain mechanisms and identification of complex neurologic overlap	Direct exploratory CPPS neuroimaging evidence plus broader chronic pain translational evidence
Tenderness	Pelvic floor myalgia, myofascial trigger points, pelvic floor	Pelvic floor dysfunction prediction, sEMG interpretation, contraction	Pelvic floor sEMG, perineal pressure signals, pelvic floor ultrasound images	More objective assessment of myofascial dysfunction and improved reproducibility of pelvic	Mostly extrapolated from mixed or female pelvic floor cohorts;

Note: Several AI applications depicted in this framework remain translational or hypothesis-generating and have not yet been clinically validated in male CPPS-specific cohorts.

**Table 2:** Main cautions and priority for future validation for artificial intelligence applications across UPOINTS domains in male chronic pelvic pain.

UPOINTS Domain	Main Interpretive Caution	Priority for Future Validation
Urinary	Urinary AI models may support CPPS phenotyping, but most were not developed in men with pelvic pain syndromes	Prospective validation in male CPPS with urinary phenotype stratification
Psychosocial	Psychosocial burden is central in CPPS, but most AI evidence reflects broader pain or mental health populations rather than disease-specific cohorts	Development of male-CPPS-specific psychosocial risk and support models
Organ-specific	These models are highly useful for exclusion of competing pathology, but they do not directly model CPPS itself	Validation as AI-supported exclusion tools within CPPS diagnostic pathways
Infection	Infection-oriented models are clinically relevant for exclusion and phenotype clarification, but do not address CPPS as a primary syndrome	Validation in men with suspected CPPS and overlapping infection-related symptoms
Neurologic	Neurologic AI is conceptually important for central sensitization phenotyping, but remains exploratory and weakly translated into routine care	Multimodal validation linking imaging, symptoms, and clinical outcomes in male CPPS
Tenderness	Pelvic floor AI appears highly relevant to the myofascial phenotype, but evidence is not yet	Male-CPPS-specific validation of pelvic floor AI tools and reference ranges

## Data Availability

No new data were created or analyzed in this study. Data sharing is not applicable to this article.
